# Sleep Matters in Chronotype and Mental Health Association: Evidence from the UK and Germany

**DOI:** 10.3390/brainsci14101020

**Published:** 2024-10-14

**Authors:** Satyam Chauhan, Kaja Faßbender, Rakesh Pandey, Ray Norbury, Ulrich Ettinger, Veena Kumari

**Affiliations:** 1 Division of Psychology, Department of Life Sciences, College of Health, Medicine and Life Sciences, Brunel University of London, Uxbridge UB8 3PH, UK; ray.norbury@brunel.ac.uk; 2 Centre for Cognitive and Clinical Neuroscience, College of Health, Medicine and Life Sciences, Brunel University of London, Uxbridge UB8 3PH, UK; 3 Department of Psychology, University of Bonn, 53113 Bonn, Germany; kaja.fassbender@uni-bonn.de (K.F.); ulrich.ettinger@uni-bonn.de (U.E.); 4 Department of Psychology, Faculty of Social Sciences, Banaras Hindu University, Varanasi 221005, India; rpan_in@yahoo.com

**Keywords:** sleep, morningness–eveningness, chronotype, mental health, personality, childhood trauma, impulsivity, schizotypy

## Abstract

Background: There is considerable evidence supporting the elevated risk of mental health problems in individuals with evening chronotype relative to those with morning or intermediate chronotypes. Recent data, however, suggest that this risk may be explained, at least partially, by poor sleep quality. Methods: This study aimed to further clarify the roles of chronotype and sleep quality in mental health outcomes (depression, anxiety, stress) in young individuals (18–40 years) living in the UK (n = 185) or Germany (n = 209). Results: Consistent with our recent observations in a comparable North Indian sample, we found that poor quality of sleep had significantly positive associations with adverse mental health outcomes both in the UK and Germany-based samples. Significant associations between evening chronotype and poor mental health were also evident, but these associations were fully mediated by poor quality of sleep in both samples. Conclusions: These observations suggest that efforts to identify sleep disruption in a timely manner and promotion of good sleep may prevent mental health problems, especially in individuals with evening chronotype and other known risks for mental disorders.

## 1. Introduction

In humans, the intra-individual variation in circadian rhythms is commonly known as ‘chronotype’ [1]. It is a multidimensional construct [2], ranging from ‘morning chronotype’ to ‘evening chronotype’, with most individuals falling in the intermediate range, known as ‘intermediate type’. Morning and evening chronotypes strongly prefer different sleep–wake timings, and the phenomenon may also impact their sleep behaviour [3]. A considerable body of evidence has shown an association between evening chronotype and various mental disorders, including depression [4,5], anxiety [6], substance-use disorder [1,7], and schizophrenia [8,9]. Of these, the most consistent association of evening chronotype has been reported to be with depression [4,5,7]. Additionally, evening chronotypes are also known to display compulsive, aggressive, and addictive behaviours, be less conscientious, have more impulsive and risky behaviour, and display negative cognitive bias, further contributing to a higher likelihood of developing mental illnesses [1,7,8].

Given that sleep timings and duration are regulated via sleep homeostatic processes [10], it is obvious to expect some form of relationship between chronotype and sleep-related disruptions. For instance, studies have shown that evening chronotypes report poor sleep quality, latency, duration, daytime dysfunction, irregular sleep–wake cycles, accumulate higher sleep debt or social jetlag, have difficulties falling and/or maintaining sleep, build higher sleep pressures, and sleep inertia [11,12,13,14,15,16]. These disturbed sleep–wake patterns have been considered to be transdiagnostic determinants for the onset and persistence of various mental health and behavioural problems, including depression and anxiety [17], psychosis [18,19], eating disorders [20], substance abuse [21], impulsive and aggressive behaviour [22], personality disorders [23], as well as mood and emotion dysregulation [24]. Both evening chronotype and poor sleep quality are found to be linked with elevated scores on psychometric measures of certain psychopathology-related personality traits, for example, neuroticism and impulsivity [1,2,3], as well as with self-reported childhood maltreatment [25]. Given these findings, there is clearly a need to better understand the influence of chronotype and sleep quality in mental health outcomes.

Against the backdrop of some studies dismissing any influence of poor sleep in chronotype-mental health association [26,27], a recent study by Muzni et al. [14] observed mental health problems to be more strongly (medium-to-large effect sizes) associated with poor sleep quality than with evening chronotype (small effect sizes) in young adults recruited from the general population in the UK (n = 675). Two very recent studies, both conducted in southeast Asian non-clinical young adult populations {North India, n = 282 [25]; HongKong, n = 200 [28]}, have also shown a strong mediating influence of sleep quality in the chronotype–mental health link. Although climate may impact chronotype [1,2,29], recent findings emerging from different parts of the world [14,25,28] question the widely reported role of chronotype as an ‘independent’ transdiagnostic risk factor for mental disorders, at least in non-clinical young adult populations.

The present study aimed to further clarify the influence of chronotype and the extent to which it might be mediated by poor sleep quality {a modifiable risk factor [17]} in mental health outcomes in a European sample (from the UK and Germany). The methods and procedures used in this study matched closely with those employed in our recent study [25]. We hypothesised, based on our recent observations in a comparable North Indian sample [25], that there will be a stronger relationship between sleep quality and mental health than between chronotype and mental health, and that any relationship between chronotype and mental health will be mediated via sleep quality. The possible influence of neuroticism, impulsivity, schizotypal personality traits, and adverse childhood experiences [30] in the chronotype–mental health association were also explored.

## 2. Methodology

### 2.1. Participants

The study involved 460 young healthy adults (aged 18–40 years) who resided in the UK (n = 213) or Germany (n = 247) at the time of their participation. Of 460, 394 participants provided usable data (UK: 185; Germany: 209). Power analysis for multiple linear regression with eight predictors, including chronotype, quality of sleep, and relevant personality traits {as in [25]}, in G*Power [31], using an alpha of 0.01, a power of 0.90, and a medium effect size (0.15), based on our recent observations [25], indicated that we required 179 participants to test our hypothesis. We aimed to recruit a minimum of 200 participants in the UK and 200 in Germany to allow sufficient power to probe our hypothesis across and within these countries.

All included participants met the study inclusion criteria of (i) being aged between 18 and 40 years (ii) being a UK/Germany resident and a native or proficient English/German speaker, (iii) not taking any regular medication (bar contraceptives and multivitamins), and (iv) having no current or previous diagnosis of a mental disorder and/or drug abuse. Of the 213 non-clinical adults assessed in the UK, 28 were excluded because they either failed our (online) attention checks [i.e., provided an answer that differed by two or more rating points for the same (duplicated) questions; (n = 26)] or did not fully complete all study measures (n = 2). Of the 247 non-clinical adults assessed in Germany, 38 were excluded for failing our attention checks. The final study sample consisted of 185 UK residents (86 males, 99 females) and 209 Germany residents (67 males, 142 females).

The study was approved by the College of Health, Medicine, and Life Science Research Ethics Committee, Brunel University of London (ref no. 36745-MHR-May/2022-39617-2), and the Research Ethics Committee of the Department of Psychology at the University of Bonn (ref no. 23-03-14). All participants signed an online consent form prior to their participation. All UK-based participants were compensated with a GBP 5 Amazon gift voucher for their time to complete the survey, while those recruited in Germany were enrolled in a lottery system for winning EUR 50.

### 2.2. Assessment of Chronotype, Mental Health, Sleep Quality, Personality Traits and Childhood Trauma

#### 2.2.1. Chronotype

The 19-item self-report Morningness–Eveningness Questionnaire (MEQ) [32] was used to assess chronotype in the UK-based sample, and its German version [33] in Germany-based sample. The questionnaire has 12 items which are rated on a Likert scale (e.g., item 6: how hungry would you be during the first hour of waking-up?), and the remaining seven items are rated on a time scale (e.g., item 1: approximately at which hour would you wake up if you were free to plan your day?). Higher MEQ scores indicate higher preference for morningness. The MEQ has been reported to have high internal consistency {*a* = 0.83 [32]}, as was also the case in our study (*a* = 0.82 and 0.87 in the UK and German-based samples, respectively).

#### 2.2.2. Mental Health

The Depression, Anxiety and Stress Scale (DASS-21) [34] was used to assess mental health in the UK-based sample and its German version [35] in Germany-based sample. The DASS-21 has three subscales: Depression, Anxiety, Stress. Each subscale consists of 7 items which are rated by the participants according to their feelings over the past one week (possible score range on each scale: 0–42). Higher scores indicate higher levels of Depression, Anxiety or Stress. Previous studies have indicated high internal consistency for all three DASS-21 subscales {Depression, *a* = 0.83–0.94; Anxiety, *a* = 0.66–0.87; Stress, *a* = 0.79–0.91 [36]}. Cronbach’s alphas in the current samples for Depression (UK, *a* = 0.89; Germany, *a* = 0.85), Anxiety (UK, *a* = 0.83; Germany, *a* = 0.78), and Stress (UK, *a* = 0.83; Germany, *a* = 0.82) also indicated high reliability coefficients.

#### 2.2.3. Sleep Quality

The Pittsburgh Sleep Quality Index [PSQI] [37] was used to assess sleep quality in the UK-based sample, and its German version [38] in Germany-based sample. The PSQI is a 19-item self-report measure assessing seven sleep facets (i.e., sleep quality, sleep efficiency, sleep disturbance, sleep dysfunction, sleep duration, daytime dysfunction, and use of sleep medication). Participants answer each item based on their sleep habits in the past month, with higher scores indicating poor sleep quality. The scale is reported to have a high internal consistency {*a* = 0.83 [37]}. The Cronbach’s alpha coefficients in the current study were *a* = 0.73 (UK) and *a* = 0.70 (Germany).

#### 2.2.4. Personality Traits

The Eysenck Personality Questionnaire-Revised Short Form (EPQ-RS) [39] was used to assess levels of Extraversion, Neuroticism, and Psychoticism in the UK-based sample, and its German version [40] in Germany-based sample. The EPQ-RS has four 12-item subscales: Extraversion, Neuroticism, Psychoticism, and Lie (48 items in total). Higher scores indicate higher levels of Extraversion, Neuroticism, and Psychoticism. The EPQ-RS is reported to have good internal consistency {Extraversion: *a* = 0.74–0.84, Neuroticism: *a* = 0.70–0.77; bar Psychoticism: *a* = 0.33–0.52 [41]}. The Cronbach’s alphas in the current sample were similar to what has been reported in the literature for Extraversion (UK, *a* = 0.83; Germany, *a* = 0.86), Neuroticism (UK, *a* = 0.82; Germany, *a* = 0.79), and Psychoticism (UK, *a* = 0.39; Germany, *a* = 0.35).

The Oxford-Liverpool Inventory of Feelings and Emotions-Short Version (s-OLIFE) [42] was used to assess schizotypy in the UK-based sample, and its German version [43] in Germany-based sample. The s-OLIFE is a 43-item self-report measure comprising four subscales assessing levels of Unusual Experiences (12 items), Cognitive Disorganisation (11 items), Introvertive Anhedonia, and Impulsive Nonconformity (10 items each), with each item rated as ‘Yes’ or ‘No’. Higher scores indicate higher levels of schizotypy. This scale is found to have high internal consistency {*a* = 0.78–0.87 [44]}. The Cronbach’s alpha coefficients in the current sample were acceptable-to-high for Unusual Experiences (UK, *a* = 0.80; Germany, *a* = 0.69) and Cognitive Disorganisation (UK, *a* = 0.82; Germany, *a* = 0.78) and lower for Introvertive Anhedonia (UK, *a* = 0.49; Germany, *a* = 0.53) and Impulsive Nonconformity (UK, *a* = 0.55; Germany, *a* = 0.42).

The Impulsive Behaviour Scale-Short Version [45] was used to assess impulsivity in the UK-based sample, and its German version [46], with four additional Positive Urgency items {as in Keidel et al. [47]}, in Germany-based sample. It is a 20-item self-report measure assessing levels of Lack of Perseverance, Lack of Premeditation, Positive Urgency, Negative Urgency, and Sensation Seeking, with each item rated on a four-point Likert scale in English and a five-point Likert scale in German {as in Keidel et al. [47]}. Higher scores indicate higher levels of impulsivity. This scale is reported to have a high internal consistency {*a* =0.74–0.88 [45]}. The Cronbach’s alpha coefficients in the current sample were in the acceptable range for Lack of Perseverance (UK, *a* = 0.63; Germany, *a* = 0.58), Lack of Premeditation (UK, *a* = 0.76; Germany, *a* = 0.65), Sensation Seeking (UK, *a* = 0.69; Germany, *a* = 0.66), Negative Urgency (UK, *a* = 0.80; Germany, *a* = 0.67), and Positive Urgency (UK, *a* = 0.82; Germany, *a* = 0.79).

#### 2.2.5. Childhood Trauma

The Childhood Trauma Questionnaire (CTQ) [48] was used to assess childhood trauma in the UK-based sample, and its German version [49] in Germany-based sample. The CTQ is a 28-item self-report measure for assessing the history and severity of Abuse (Physical, Emotional, Sexual), Neglect (i.e., Emotional, Physical), and Denial, with each item being rated on a five-point Likert scale. Higher scores indicate severity of abuse and neglect. This scale is reported to have a high internal consistency {*α* = 0.66–0.92 [48]}. In the current sample, the Cronbach’s alpha coefficients were high for Physical Abuse (UK, *a* = 0.83; Germany, *a* = 0.83), Sexual Abuse (UK, *a* = 0.94; Germany, *a* = 0.88), Emotional Abuse (UK, *a* = 0.81; Germany, *a* = 0.83), and Emotional Neglect (UK, *a* = 0.83; Germany, *a* = 0.88), but was considerably lower for Physical Neglect (UK, *a* = 0.62; Germany, *a* = 0.42).

### 2.3. Statistical Analysis

All statistical analyses were conducted using Statistical Package for Social Sciences (SPSS) or SPSS Amos (Windows version 28; IBM, New York, NY, USA), with alpha value maintained at *p* < 0.05 unless specified otherwise.

To begin with, all data properties (skewness and kurtosis < ±2) were examined, followed by a reliability assessment of the various self-report scales. Since the Psychoticism subscale of the EPQ-RS showed poor reliability (UK, *α* = 0.39; Germany, *a* = 0.35), it was not included in any further analyses. Prior to running any statistical analyses to probe our hypothesis, we conducted a series of independent sample *t*-tests to compare the UK- and Germany-based participants on mental health, sleep, chronotype, personality traits, and childhood trauma parameters to rule out any major differences between them. Given that the UK-based sample, on average, had significantly poor mental health and sleep quality scores (as well as larger range of scores on these variables) compared to the Germany-based sample (see Section 3.1), all further analyses were conducted separately for the UK- and Germany-based samples, and then significant effects were statistically evaluated for any UK versus Germany differences. Given that chronotype may be sex-dependent [1], we also explored sex-related differences (separately in the UK- and Germany-based samples) in mental health, sleep quality, personality traits, and childhood trauma measures using a series of independent sample *t*-tests.

Pearson correlations were employed to investigate the potential relationships of chronotype (MEQ scores) with mental health, sleep quality, personality traits, and childhood trauma, as well as the relationship of sleep quality with mental health variables. We interpreted effect sizes for observed correlation coefficients (*r* values) based on the recommendations of Cohen [50] (absolute *r* value 0.1 to 0.29: small; 0.3 to 0.49: medium; 0.5 to 1: large), as in our previous study [25]. A Fisher’s Exact *z*-test was used to test for statistically significant sex-related differences in these relationships.

**Figure 1:**
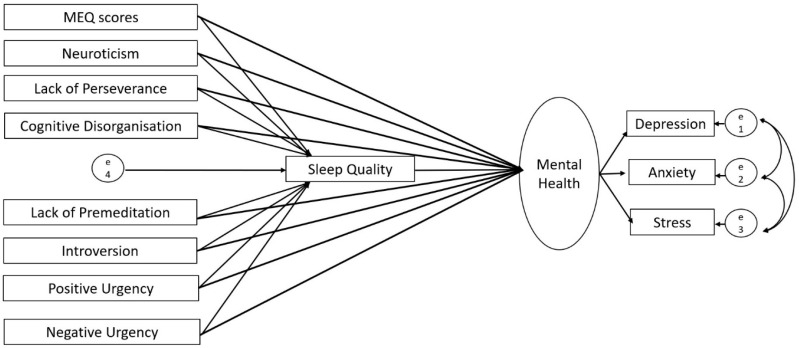
Proposed structural model displaying the direct and indirect (through sleep quality) influences of chronotype and personality traits (predictors; allowed to covary) on mental health (outcome).

**Figure 2:**
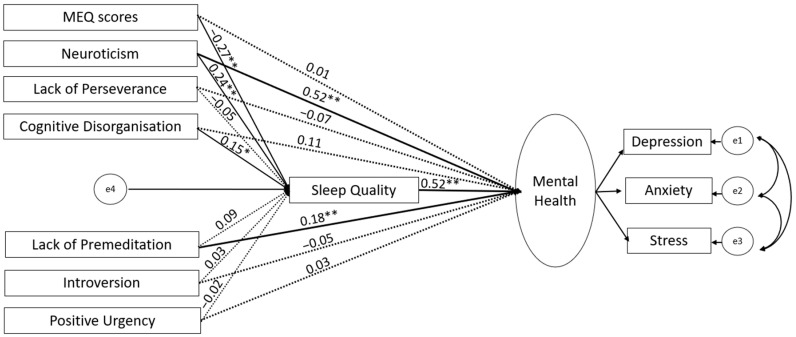
Results of the initial SEM analyses in the UK-based sample. Solid lines denote significant paths (** *p* < 0.001, * *p* < 0.005) and dotted lines denote non-significant paths.

**Figure 3:**
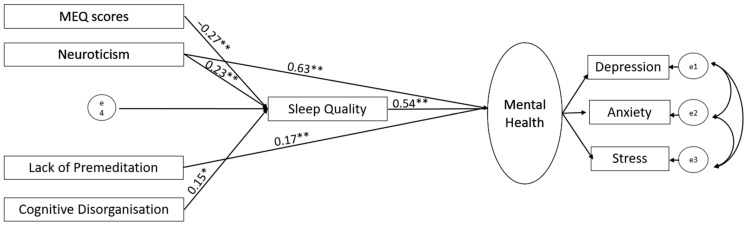
Revised (final) model displaying significant paths (** *p* < 0.001, * *p* < 0.005) in the UK-based sample.

**Figure 4:**
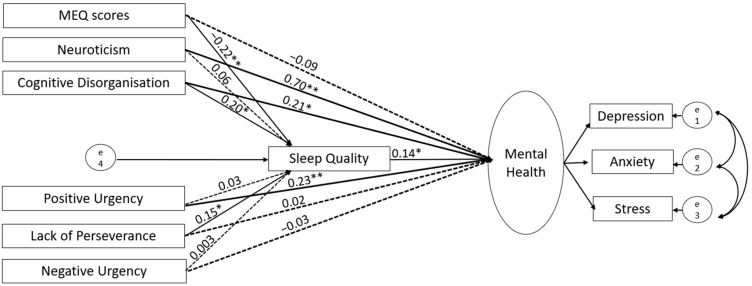
Results of the initial SEM analyses in the Germany-based sample. Solid lines denote significant paths (** *p* < 0.001, * *p* < 0.005) and dotted lines denote non-significant paths.

**Figure 5:**
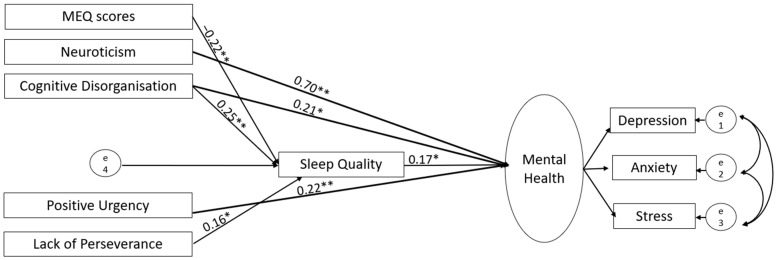
Revised (final) model displaying significant paths (** *p* < 0.001, * *p* < 0.005) in the Germany-based sample.

Based on the correlations of evening chronotype with mental health, quality of sleep, and relevant personality measures (see Section 3.2), we ran structural equation modelling (SEM) using SPSS Amos (version 28; IBM, New York, NY, USA), first in the UK and then in Germany-based sample, with chronotype and personality traits as predictors (allowed to covary), sleep quality as a mediator, and mental health (a latent construct integrating depression, anxiety and stress subscales) as an outcome (Figure 1). Following our earlier study [25], we used the maximum likelihood method to assess model parameters. A good model fit was based on the following criteria: (a) comparative fit index (CFI) > 0.95, (b) root mean square error of approximation (RMSEA) < 0.08, (c) ratio of maximum-likelihood chi-square to the degree of freedom (χ^2^/*df*) < 5, (d) goodness of fit index (GFI) > 0.95, (e) adjusted goodness of fit index (AGFI) > 0.90, (f) and Tucker–Lewis Index (TLI) > 0.95 [51]. We tested the statistical significance of direct and indirect paths using a bias-corrected 95% bootstrap confidence interval and corresponding *p* values. After testing our initially proposed model (Figure 1), first in the UK and then in Germany, we revised it by removing all non-significant paths (UK, Figure 2 and Figure 3; Germany, Figure 4 and Figure 5; reproduced in Microsoft Power Point, Windows version 2019 based on SPSS Amos generated outputs). Lastly, to explore any sex-related differences, we compared the fully constrained model (measurement weights of the measurement model of mental health, structural weights, covariances and residuals constrained to be equal in males and females) with the unconstrained model. A non-significant chi-square difference (*p* > 0.05), ΔCFI ≤ 0.005, and ΔRMSEA ≤ 0.01 indicated invariance [52,53]. A similar approach was taken to examine country (UK versus Germany)-related differences.

## 3. Results

### 3.1. Sample Characterisation

The demographic characteristics of the UK and German-based samples are presented in Table 1. Overall, the UK-based sample scored higher than the Germany-based sample on Depression (*t_391_ =* 4.00, *p* < 0.001), Anxiety (*t_391_ =* 5.18, *p* < 0.001), Stress (*t_391_ =* 2.69, *p* = 0.004), Neuroticism (*t_391_ =* 5.23, *p* < 0.001), Unusual Experiences (*t_391_ =* 8.32, *p* < 0.001), Cognitive Disorganisation (*t_391_ =* 4.05, *p* < 0.001), Introvertive Anhedonia (*t_391_ =* 7.19, *p* < 0.001), Emotional Abuse (*t_391_ =* 4.15, *p* < 0.001), Physical Abuse (*t_386_ =* 6.43, *p* < 0.001), Sexual Abuse (*t_390_ =* 5.85, *p* < 0.001), Emotional Neglect (*t_389_ =* 4.12, *p* < 0.001), Physical Neglect (*t_391_ =* 5.22, *p* < 0.001), Negative Urgency (*t_391_ =* 2.99, *p* = 0.001), Sensation Seeking (*t_391_ =* 5.57, *p* < 0.001), and Positive Urgency (*t_391_ =* 6.04, *p* < 0.001), and also rated themselves as having poor sleep quality (*t_391_* = 3.30, *p* < 0.001). The Germany-based sample scored higher on Lack of Perseverance (*t_391_ =* 30.02, *p* < 0.001) and Lack of Premeditation (*t_391_ =* 25.77, *p* < 0.001).

In the UK-based sample, on average, females were younger than males (*t_183_* = 2.03, *p* = 0.022) and scored higher on Anxiety (*t_183_* = 2.45, *p* = 0.007), Stress (*t_183_* = 3.28, *p* < 0.001), Neuroticism (*t_183_* = 5.13, *p* < 0.001), Sleep Quality (*t_183_* = 2.56, *p* = 0.006), Unusual Experiences (*t_183_* = 2.26, *p* = 0.012), Cognitive Disorganisation (*t_183_* = 3.80, *p* < 0.001), Negative Urgency (*t_183_* = 2.12, *p* = 0.017), and Emotional Abuse (*t_183_* = 2.28, *p* = 0.012), while males scores higher in Sensation Seeking (*t_183_* = 2.38, *p* = 0.009) (Table 2).

In the Germany-based sample, females, on average, had higher scores than males for Stress (*t_207_* = 2.36, *p* = 0.009), Neuroticism (*t_207_* = 3.66, *p* < 0.001), Sleep Quality (*t_207_* = 2.78, *p* = 0.003), Unusual Experiences (*t_207_* = 1.74, *p* = 0.041), Cognitive Disorganisation (*t_207_* = 2.77, *p* = 0.003), and Emotional Abuse (*t_207_* = 2.09, *p* = 0.019), while males scored higher for Sensation Seeking (*t_207_* = 3.06, *p* < 0.001) and Negative Urgency (*t_207_* = 1.98, *p* = 0.024) (Table 2).

### 3.2. Associations between Chronotype, Sleep Quality, Mental Health, Personality Traits, and Childhood Trauma

#### 3.2.1. UK

Evening chronotype, indicated by lower MEQ scores, was associated with higher levels of Depression (*r* = −0.242, *p* < 0.001) and higher Extraversion scores (*r* = 0.226, *p* = 0.002). Evening chronotype was also correlated significantly with higher BMI (*r* = −0.227, *p* = 0.002). While some correlations appeared numerically stronger in females than males, none of the correlation differences were statistically significant (*p* > 0.05) (see Table 3).

As expected, poor sleep quality, indicated by higher PSQI scores, was correlated with higher levels of Depression (*r* = 0.565, *p* < 0.001), Anxiety (*r* = 0.535, *p* < 0.001), Stress (*r* = 0.510, *p* < 0.001); higher scores on psychopathology-related personality traits, including Neuroticism (*r* = 0.379, *p* < 0.001), Unusual Experiences (*r* = 0.236, *p* < 0.001), Cognitive Disorganisation (*r* = 0.363, *p* < 0.001), Introvertive Anhedonia (*r* = 0.175, *p* = 0.017), Impulsive Nonconformity (*r* = 0.203, *p* = 0.006), Negative Urgency (*r* = 0.315, *p* < 0.001), and Positive Urgency (*r* = 0.175, *p* = 0.017); and also with Emotional Abuse (*r* = 0.422, *p* < 0.001) and Sexual Abuse (*r* = 0.230, *p* = 0.002) (see Supplementary Table S1). Poor sleep quality also correlated with evening chronotype (i.e., lower MEQ scores) (*r* = −0.296, *p* < 0.001), and although this correlation seemed numerically stronger in males than in females, the correlation difference was not statistically significant (*p* > 0.05). Overall, chronotype had small-sized correlations with mental health outcomes, whereas sleep quality had large sized correlations with mental health outcomes. 

#### 3.2.2. Germany

In line with the UK findings, evening chronotype was significantly associated with Depression (*r* = −0.299, *p* < 0.001) and Stress (*r* = −0.234, *p* < 0.001), as well as with higher levels of Neuroticism (*r =* −0.206, *p* = 0.003), Cognitive Disorganisation (*r =* −0.302, *p* < 0.001), Negative Urgency (*r =* −0.147, *p* = 0.033), and Lack of Premeditation (*r =* 0.159, *p* = 0.022). Again, some correlations appeared to be numerically stronger in females than in males, but none of these differences were formally significant (*p* > 0.05) (see Table 3).

As expected, poor sleep quality correlated with Depression (*r* = 0.275, *p* < 0.001), Anxiety (*r* = 0.305, *p* < 0.001), and Stress (*r* = 0.271, *p* < 0.001), the personality traits of Neuroticism (*r* = 0.244, *p* < 0.001), Unusual Experiences (*r* = 0.308, *p* < 0.001), Cognitive Disorganisation (*r* = 0.289, *p* < 0.001), Introvertive Anhedonia (*r* = 0.196, *p* = 0.005), Impulsive Nonconformity (*r* = 0.186, *p* = 0.007), and Negative Urgency (*r* = 0.157, *p* = 0.024), and also with Emotional Abuse (*r* = 0.261, *p* < 0.001), Physical Abuse (*r* = 0.181, *p* = 0.010), and Sexual Abuse (*r* = 0.188, *p* = 0.007) (see Supplementary Table S2). Poor sleep quality also correlated with evening chronotype (*r* = −0.276, *p* < 0.001); and, again, although this correlation seemed numerically stronger in females relative to males, the correlation difference was not statistically significant (*p* > 0.05). Overall, both chronotype and sleep quality had small-to-medium-sized correlations with mental health outcomes.

### 3.3. The Mediating Role of Sleep Quality: SEM Analysis

#### 3.3.1. UK

Our proposed model (Figure 2) had a good fit to the data (χ^2^/*df* = 1.13, *p* < 0.001; RMSEA = 0.01; GFI = 0.96; AGFI = 0.90; CFI = 0.99) but had a poor local fit. We therefore revised it by removing non-significant paths to reach our final model (χ^2^/*df* = 0.99; GFI = 0.97; TLI = 1; CFI = 1; RMSEA = 0.000) (see Figure 3). As evident in Figure 3, there was no significant direct influence of chronotype on mental health; instead, the chronotype–mental health relationship was fully mediated by poor sleep quality. The mental health relationship with Neuroticism and Cognitive Disorganisation was also partially mediated by poor sleep quality. Lastly, we found no sex-related influence in the final model, as indicated by non-significant differences [Δχ^2^(8) = 4.68, *p* = 0.791; ΔCFI = 0; ΔRMSEA = 0.008] when comparing the model fit of the unconstrained model with that of the structural-weight-constrained model.

#### 3.3.2. Germany

In line with the UK findings, our proposed model (Figure 4) had an acceptable fit (χ^2^/*df* = 4.38, *p* < 0.001; RMSEA = 0.12; GFI = 0.94; AGFI = 0.78; CFI = 0.92) to the data, but it was revised, due to poor local fit, to remove the non-significant paths (model fit indices: χ^2^/*df* = 3.72; GFI = 0.93; TLI = 0.82; CFI = 0.91; RMSEA = 0.11) (see Figure 5). As depicted in Figure 5, we found no direct effect of chronotype on mental health and observed that its relationship with mental health was fully mediated by poor sleep quality. We also found that sleep quality partially mediated the association of mental health with Cognitive Disorganisation and Lack of Perseverance. While exploring sex differences, we found that the comparison of the unconstrained model with the structural-weight-constrained model showed a non-significant chi-square difference Δχ^2^(9) = 16.44, *p* = 0.058 and RMSEA (ΔRMSEA = 0.003) but a significant difference in CFI (ΔCFI = 0.013). The pairwise difference in the path coefficients of the unconstrained model in males and females showed a significant difference in the path linking sleep quality with mental health [Critical ratio = 2.04; stronger in females (β = 0.252) than males (β = 0.028)], and the path linking Cognitive Disorganisation with sleep quality [Critical ratio = 2.10; stronger in males (β = 0.485) than females (β = 0.128)], suggesting a partial variance in the model.

#### 3.3.3. Chronotype, Sleep Quality, and Mental Health Associations: UK versus Germany

When exploring the possible invariance of the path model across the UK and Germany-based samples, we found the measurement model of mental health to be variant [Δχ^2^(2) = 11.22, *p* = 0.004; ΔCFI = 0.008; ΔRMSEA = 0.013]. The factor loading of anxiety in the UK (β = 0.675) and Germany (β = 0.531) was found to be significantly different (Critical ratio = 3.36). Additionally, compared to the measurement-weight-constrained model, the structural-weight-constrained model also differed [Δχ^2^(6) = 18.73, *p* = 0.005; ΔCFI = 0.011] although with a non-significant RMSEA (ΔRMSEA = 0.005). The pairwise difference in the path coefficients of the measurement-weight-constrained model showed a difference in the path linking sleep quality to mental health (CR = 3.84), this being stronger in the UK (β = 0.55) than Germany (β = 0.16).

## 4. Discussion

The present study aimed to further examine our recent finding of sleep quality as a mediating factor in the chronotype–mental health relationship in young non-clinical (healthy) adults residing in North India [25] in a sample of young non-clinical adults residing in the UK or Germany while also quantifying the role of psychopathology-related personality traits and childhood trauma in this relationship. Unexpectedly, our UK (London)-based participants, on average, were found to have, higher levels of depression, anxiety, and stress, as well as poor sleep quality, compared to those who were residing in Germany (Bonn). This may be related to a difference in the recruitment strategy used in the UK and Germany. In the UK, each recruited participant received GBP 5 for their participation, while those recruited in Germany were enrolled in a lottery system to win EUR 50. A small but guaranteed financial incentive that was offered to each participant in the UK might have attracted more participants belonging to a lower socioeconomic background which is known to be associated with poor mental health and reduced psychological well-being [54,55,56].

In relation to our study hypothesis, the key findings of the present study were: (i) Evening chronotype (lower MEQ scores) had small-to-medium-sized associations with metal health outcomes (UK and Germany, *r* values: 0.20–0.30), (ii) Poor sleep quality had large associations with mental health outcomes in the UK-based sample (*r* values: 0.51–0.56), while small-to-medium-sized associations were observed in Germany-based sample (*r* values: 0.27–0.30). (iii) Sleep quality fully mediated the chronotype–mental health relationship, with no significant direct effect of evening chronotype on mental health outcomes in either the UK- or Germany-based samples. Evening chronotype had significant but mostly small-to-medium-sized (*r* values, 0.14–0.34) associations with psychopathology-relevant personality traits in both samples. The association between evening chronotype and severity of childhood emotional maltreatment, although in line with our earlier findings in the North Indian sample [25], was not formally significant in the UK- or Germany-based samples.

In the present study, we employed same methods and replicated our previous findings in a North Indian sample [25] in showing that sleep quality fully mediated the chronotype-mental health association in non-clinical young UK and Germany-based samples, though this effect was weaker in Germany-based sample, possibly due to a limited range of scores on measures of both mental health and sleep (Table 2) as well as a possible difference between the UK and Germany-based samples in resilience that was recently reported to impact both chronotype–mental health and sleep–mental health associations [28]. Nonetheless, our findings across India, the UK, and Germany are generally in line with previous correlational studies that have consistently found an association between evening chronotype and depressive symptoms [4,5] as well as general mental health [57]. Some longitudinal studies show that the prevalence of higher levels of depression predicts evening chronotype, especially in adolescents [58,59], but there are also some longitudinal studies, using actigraphy, that failed to detect an association between depression and evening chronotype in adolescents [60,61]. These studies, however, did not consider sleep-related disturbances, including poor sleep latency, quality, and duration, all of which are known to be more common in evening chronotypes [13,14,25,28], as also shown in the current study. The mediating role of sleep quality in the chronotype–mental health relationship is also visible in clinically depressed individuals [62]. Further support for the mediating role of sleep quality in the chronotype–poor mental health link comes from recent findings suggesting that this link is either attenuated or absent in the presence of sufficient and good quality sleep (for example, in individuals who can work remotely) in evening chronotypes [63].

In the modern world, humans realistically rely less on their internal clock and more on the social clock to sleep, which disrupts and shifts their circadian rhythms [64,65] of melatonin and cortisol secretions [66,67], both linked with psychiatric illnesses such as schizophrenia and depression [68,69]. One of the most noticeable forms of circadian disruption is sleep disturbance and social jetlag, commonly found in evening chronotypes [14,65,70] due to their natural tendency to be awake at later hours, which causes difficulties in sleep restoration and falling asleep [3,71]. Not surprisingly, studies have reported insomnia severity (β = −0.14) as a significant moderator of the chronotype–mental health relationship [57]. Taken together, non-restoration of sleep and/or poor sleep habits as a result of disrupted circadian rhythms may explain previously observed positive associations between evening chronotype and adverse mental health outcomes. The prevalence of poor sleep quality may render evening chronotypes more susceptible to developing mental health issues. This may be especially true for people who have lower resilience [28,72], though such a possibility was not directly addressed in the current study.

While investigating the influence of psychopathology-related traits, we found a small-sized positive association between evening chronotype and neuroticism in female participants of both the UK and Germany-based samples. This is consistent with previous findings on this topic [1,2,3,14]. Interestingly, this relationship was somewhat weaker and non-significant for males, who also scored, on average, lower than females, which is not surprising given known sex differences in neuroticism (females > males) across countries and cultures [73]. Extraversion had a small association with morning chronotype in the UK, which is also consistent with the previous literature [3,14,25]. This relationship, however, was not found in Germany-based sample for reasons that we do not fully understand. There are some other studies that have found no significant associations between extraversion and morning chronotype [1]. We found a small correlation between evening chronotype and impulsivity both in the UK and German-based samples. This has also been seen in previous studies [25,74]. Impulsivity as a personality trait has been linked with impulsive behaviour in healthy and clinical populations [75] and might explain why evening chronotypes may be more likely to engage in substance abuse and addiction [1]. Interestingly, we also replicated our previous findings of a small but significant association between cognitive disorganisation aspect of schizotypy and evening chronotype in both UK (females) and German (all) participants. Individuals scoring high on schizotypy share some characteristics with schizophrenia patients [76], including higher stress-reactivity and anxiety [77,78,79], which disrupts sleep cycles [80], and sleep deprivation in turn can induce psychosis-like symptoms in healthy adults [19,81,82].

### 4.1. Limitations and Future Directions

The study had some limitations. First, we used self-report questionnaires and did not control for light exposure, and menstrual cycle phase in females, both of which may influence sleep and mental health [1,83]. Second, we restricted our sample to young adults (≤40 years), and thus the findings cannot be generalised to adolescents (≤17 years) or older adults (>40 years). Third, our study used chronotype as continuous variable and employed a cross-sectional design; therefore, it cannot speak of causation. Further studies employing objective measures of circadian rhythm alongside relevant self-report measures in a longitudinal design and different age groups are needed to substantiate and refine the present findings.

### 4.2. Conclusions

To conclude, we did not observe any direct impact of chronotype on mental health; instead, this association was found to be fully mediated by poor sleep quality in young adults living in the UK or Germany. These and our previous findings [25] argue against the independent role of chronotype as a transdiagnostic risk factor for mental health problems in non-clinical young adults and highlight sleep disruption and circadian misalignment as important therapeutic targets for improving mental health outcomes. Intervening early on to ensure good sleep quality may be a preventive strategy in combination with attempts to shift circadian preference towards morning.

## Figures and Tables

**Table 1:** Demographic characteristics of the UK and Germany-based participants.

		UK	Germany
		Frequency (%) of N = 185	Frequency (%) of N = 209
Ethnicity	White European	30.3%	79.4%
	Any Other White	0%	1%
	South Asian	46.5%	6.7%
	East Asian	3.8%	1.9%
	West Asian	0.5%	1.4%
	Mixed	7.6%	4.3%
	Black	7%	0.5%
	Other Ethnicities	4.3%	3.8%
	Prefer Not to Say	0%	1%
Stimulant/Sedative Consumption ^a^	Caffeine	39.5%	-
	Nicotine	49.2%	-
	Alcohol	7%	-
	Others	2.7%	-
	Prefer Not to Say	1.6%	-
BMI ^b^	Underweight	39.5%	42.1%
	Normal	45.4%	52.6%
	Overweight	8.6%	4.8%
	Obese	3.2%	0.5%
Education/Employment	Student	73.5%	95.2%
	Full-time	26.5	4.8%
Sleep Quality ^c^	Good (≤5)	57.8%	65.6%
	Poor (6–14)	42.2%	34.4%

Abbreviation: BMI, Body Mass Index. Underweight (<18.5), Normal Weight (18.5–24.9), Overweight (25–24.9), Obese (>30). ^a^ Data not collected in the Germany-based sample. ^b^ BMI data missing for six participants in the UK. ^c^ Score 0–5: good sleepers; score 6 and above: poor sleepers.

**Table 2:** Descriptive statistics for chronotype, mental health, sleep quality, personality traits, and childhood trauma measures.

Study Variables			UK						Germany					
			Males (*n* = 86)		Females (*n* = 99)		All (N = 185)		Males (*n* = 67)		Females (*n* = 142)		All (N = 209)	
			Mean ± SD	Sample Range	Mean ± SD	Sample Range	Mean ± SD	Sample Range	Mean ± SD	Sample Range	Mean ± SD	Sample Range	Mean ± SD	Sample Range
Age			25.13 ± 5.27	18–39	23.77 ± 3.77	18–38	24.41 ± 4.57	18–39	23.81 ± 4.12	18–36	23.05 ± 3.39	18–38	23.29 ± 3.65	18–38
Chronotype	MEQ		47.59 ± 8.89	21–68	49.68 ± 11.05	27–78	48.71 ± 10.13	21–78	49.19 ± 10.71	22–74	50.04 ± 9.73	22–71	49.77 ± 10.04	22–74
Mental Health	DASS-21	D	10.23 ± 9.62	0–42	12.57 ± 10.77	0–42	11.48 ± 10.29	0–42	8.03 ± 8.66	0–38	7.76 ± 7.07	0–36	7.85 ± 7.60	0–38
		A	8.74 ± 8.26	0–40	12.02 ± 9.67	0–40	10.50 ± 9.16	0–40	5.46 ± 6.21	0–34	6.59 ± 7.33	0–34	6.23 ± 7.00	0–34
		S	10.72 ± 8.14	0–32	15.05 ± 9.58	0–38	13.04 ± 9.18	0–38	8.78 ± 8.31	0–38	11.58 ± 7.83	0–34	10.68 ± 8.08	0–38
Quality of Sleep	PSQI	SQ	1.02 ± 0.61	0–2	1.17 ± 0.59	0–3	1.10 ± 0.60	0–3	0.96 ± 0.58	0–2	1.02 ± 0.53	0–3	1.00 ± 0.55	0–3
		SL	1.30 ± 0.97	0–3	1.41 ± 0.93	0–3	1.36 ± 0.95	0–3	1.01 ± 0.80	0–3	1.15 ± 0.88	0–3	1.11 ± 0.86	0–3
		SD	0.73 ± 0.78	0–3	0.78 ± 0.73	0–3	0.75 ± 0.75	0–3	0.08 ± 0.26	0–1	0.20 ± 0.48	0–2	0.16 ± 0.43	0–2
		SE	0.56 ± 0.91	0–3	0.77 ± 1.05	0–3	0.67 ± 0.99	0–3	0.32 ± 0.53	0–2	0.47 ± 0.74	0–3	0.42 ± 0.68	0–3
		SDis	1.05 ± 0.44	0–3	1.30 ± 0.50	0–2	1.18 ± 0.49	0–3	0.92 ± 0.40	0–2	1.04 ± 0.38	0–3	1.00 ± 0.39	0–3
		SMed	0.03 ± 0.18	0–1	0.16 ± 0.48	0–3	0.10 ± 0.38	0–3	0.01 ± 0.12	0–1	0.06 ± 0.34	0–3	0.05 ± 0.29	0–3
		DDys	1.05 ± 0.83	0–3	1.26 ± 0.82	0–3	1.16 ± 0.83	0–3	1.07 ± 0.70	0–2	1.25 ± 0.67	0–3	1.20 ± 0.69	0–3
		Global Score	5.21 ± 2.31	0–11	6.12 ± 2.50	1–14	5.70 ± 2.45	0–14	4.35 ± 1.76	0–8	5.21 ± 2.20	0–13	4.94 ± 2.11	0–13
Personality Traits	EPQ	Extrav	6.71 ± 3.40	0–12	7.40 ± 3.41	0–12	7.08 ± 3.41	0–12	7.93 ± 3.70	0–12	7.31 ± 3.49	0–12	7.51 ± 3.56	0–12
		Neuro	5.57 ± 3.44	0–12	7.99 ± 2.96	2–12	6.86 ± 3.40	0–12	4.01 ± 3.21	0–12	5.66 ± 2.93	0–12	5.13 ± 3.11	0–12
	s-OLIFE	UnEx	4.62 ± 3.10	0–12	5.68 ± 3.22	0–11	5.19 ± 3.20	0–12	2.44 ± 2.31	0–10	3.04 ± 2.28	0–9	2.83 ± 2.31	0–10
		CogDis	5.20 ± 3.25	0–11	6.96 ± 3.03	0–11	6.15 ± 3.25	0–11	4.01 ± 2.91	0–11	5.24 ± 3.03	0–11	4.82 ± 3.05	0–11
		IntroAn	3.19 ± 1.95	0–8	3.35 ± 1.94	0–8	3.28 ± 1.94	0–8	1.97 ± 1.76	0–8	1.96 ± 1.70	0–9	1.95 ± 1.72	0–9
		ImpNn	2.75 ± 1.92	0–8	3.07 ± 2.05	0–8	2.92 ± 1.98	0–8	2.79 ± 1.73	0–7	2.64 ± 1.73	0–7	2.67 ± 1.74	0–7
	S-UPPS-P	NegU	8.94 ± 3.04	4–16	9.90 ± 3.11	4–16	9.45 ± 3.11	4–16	8.19 ± 2.50	4–16	8.85 ± 2.10	4–13	8.64 ± 2.25	4–16
		LackP	6.55 ± 1.77	4–11	6.89 ± 2.01	4–14	6.74 ± 1.91	4–14	12.05 ± 1.64	9–16	12.29 ± 1.72	7–16	12.22 ± 1.69	7–16
		LackPre	6.67 ± 2.27	4–15	7.14 ± 2.06	4–13	6.92 ± 2.17	4–15	12.09 ± 1.63	8–16	11.82 ± 1.66	8–16	11.91 ± 1.65	8–16
		SenS	11.89 ± 2.79	5–16	10.90 ± 2.82	5–16	11.36 ± 2.84	5–16	10.61 ± 2.71	5–16	9.45 ± 2.47	4–16	9.82 ± 2.60	4–16
		PosU	8.30 ± 3.19	4–16	8.66 ± 3.04	5–16	8.49 ± 3.11	4–16	7.18 ± 2.52	4–15	6.67 ± 2.19	4–12	6.83 ± 2.31	4–15
Childhood Trauma	CTQ-SF	EAb	9.14 ± 3.91	5–22	10.67 ± 5.02	5–25	9.96 ± 4.59	5–25	7.38 ± 3.49	5–23	8.55 ± 3.88	5–25	8.18 ± 3.79	5–25
		PAb	7.73 ± 3.62	5–19	7.62 ± 4.17	5–24	7.67 ± 3.91	5–24	5.42 ± 1.15	5–11	5.74 ± 2.39	5–24	5.64 ± 2.08	5–24
		SAb	7.02 ± 4.32	5–21	8.02 ± 5.51	5–25	7.56 ± 5.00	5–25	5.14 ± 1.00	5–13	5.50 ± 1.93	5–22	5.38 ± 1.69	5–22
		ENeg	10.87 ± 4.46	5–25	11.41 ± 4.6	5–23	11.16 ± 4.53	5–25	8.90 ± 4.02	5–19	9.49 ± 4.34	5–25	9.30 ± 4.24	5–25
		PNeg	8.35 ± 3.20	5–17	8.00 ± 3.19	5–18	8.16 ± 3.19	5–18	6.67 ± 2.21	5–16	6.69 ± 2.42	5–17	6.68 ± 2.35	5–17

Abbreviations: MEQ: Morningness–Eveningness Questionnaire; DASS-21: Depression Anxiety and Stress Scale-21 (subscales: D, Depression; A, Anxiety; S, Stress); PSQI, Pittsburgh Sleep Quality Index (sleep facets: DDys, Daytime Dysfunction; SD, Sleep Duration; SDis, Sleep Disturbance; SE, Sleep Efficiency; SL, Sleep Latency; SMed, Sleep Medication; SQ, Sleep Quality); EPQ-SF, Eysenck Personality Questionnaire-Revised (subscales: Extrav, Extraversion; Neuro, Neuroticism); s-OLIFE, short Oxford-Liverpool Inventory of Feelings and Emotions (subscales: UnEx, Unusual Experience; CogDis, Cognitive Disorganisation; IntroAn, Introvertive Anhedonia; ImpNn, Impulsive Nonconformity); S-UPPS-P, Impulsive Behaviour Scale-Short Version (subscales: NegU, Negative Urgency; LackP, Lack of Perseverance; LackPre, Lack of Premeditation; SenS, Sensation Seeking; PosU, Positive Urgency); CTQ-SF, short form of Childhood Trauma Questionnaire (subscales: EAb, Emotional Abuse; PAb, Physical Abuse; SAb, Sexual Abuse; ENeg, Emotional Neglect; PNeg, Physical Neglect). Note: Physical abuse data missing for four participants and sexual and emotional abuse data missing for one participant (all German females).

**Table 3:** Chronotype (MEQ) associations (Pearson’s *r*) with mental health, quality of sleep, personality traits, and childhood trauma.

	Scales	Variables	Chronotype (MEQ Scores)					
			UK			Germany		
			Males	Females	All	Males	Females	All
Mental Health	DASS-21	D	−0.183	−0.304	−0.242	−0.270	−0.317	−0.299
			(0.091)	(0.002)	(<0.001)	(0.027)	(<0.001)	(<0.001)
		A	−0.059	−0.207	−0.130	−0.139	−0.131	−0.129
			(0.590)	(0.040)	(0.077)	(0.262)	(0.119)	(0.062)
		S	−0.046	−0.179	−0.101	−0.324	−0.200	−0.234
			(0.675)	(0.077)	(0.172)	(0.008)	(0.017)	(<0.001)
Quality of Sleep	PSQI	SQ	−0.349	−0.307	−0.296	−0.227	−0.320	−0.276
			(0.001)	(0.002)	(<0.001)	(0.067)	(<0.001)	(<0.001)
Personality Traits	EPQ-SF	Extrav	0.102	0.301	0.226	0.154	−0.004	0.049
			(0.349)	(0.002)	(0.002)	(0.214)	(0.966)	(0.485)
		Neuro	0.013	−0.239	−0.079	−0.220	−0.225	−0.206
			(0.908)	(0.017)	(0.287)	(0.073)	(0.007)	(0.003)
	s-OLIFE	UnEx	0.205	−0.066	0.059	−0.099	−0.130	−0.113
			(0.058)	(0.514)	(0.426)	(0.424)	(0.124)	(0.102)
		CogDis	−0.028	−0.238	−0.112	−0.342	−0.303	−0.302
			(0.799)	(0.018)	(0.128)	(0.005)	(<0.001)	(<0.001)
		IntroAn	0.084	0.028	0.055	−0.193	−0.012	−0.075
			(0.443)	(0.782)	(0.461)	(0.119)	(0.888)	(0.282)
		ImpNn	0.028	−0.103	−0.043	−0.141	−0.125	−0.132
	S-UPPS-P		(0.797)	(0.310)	(0.562)	(0.255)	(0.137)	(0.057)
		NegU	0.063	−0.117	−0.027	−0.162	−0.150	−0.147
			(0.565)	(0.247)	(0.713)	(0.191)	(0.076)	(0.033)
		LackP	0.030	−0.253	−0.135	0.213	0.130	0.159
			(0.783)	(0.011)	(0.067)	(0.084)	(0.124)	(0.022)
		LackPre	0.019	−0.203	−0.093	−0.043	0.101	0.049
			(0.863)	(0.044)	(0.210)	(0.727)	(0.233)	(0.481)
		SenS	−0.043	0.051	−0.005	0.048	−0.003	0.007
			(0.694)	(0.616)	(0.941)	(0.700)	(0.967)	(0.925)
		PosU	0.063	−0.204	−0.084	−0.292	0.026	−0.096
			(0.566)	(0.043)	(0.255)	(0.016)	(0.756)	(0.167)
Childhood Trauma	CTQ-SF	EAb	−0.066	−0.170	−0.113	−0.079	−0.073	−0.068
			(0.549)	(0.093)	(0.125)	(0.526)	(0.389)	(0.328)
		PAb	0.109	−0.120	−0.035	−0.150	0.026	−0.007
			(0.316)	(0.236)	(0.637)	(0.228)	(0.761)	(0.923)
		Sab	0.031	0.039	0.046	−0.039	0.135	0.097
			(0.780)	(0.701)	(0.535)	(0.752)	(0.109)	(0.163)
		ENeg	−0.051	−0.096	−0.071	0.025	−0.002	0.008
			(0.639)	(0.343)	(0.336)	(0.842)	(0.982)	(0.906)
		PNeg	0.190	0.010	0.078	0.231	0.041	0.101
			(0.079)	(0.919)	(0.292)	(0.060)	(0.631)	(0.145)

Abbreviations: DASS-21: Depression Anxiety and Stress Scale-21 (subscales: D, Depression; A, Anxiety; S, Stress); PSQI, Pittsburgh Sleep Quality Index (SQ, Sleep Quality); EPQ-SF, Eysenck Personality Questionnaire-Revised (subscales: Extrav, Extraversion; Neuro, Neuroticism); s-OLIFE, short Oxford-Liverpool Inventory of Feelings and Emotions (subscales: UnEx, Unusual Experience; CogDis, Cognitive Disorganisation; IntroAn, Introvertive Anhedonia; ImpNn, Impulsive Nonconformity); S-UPPS-P, Impulsive Behaviour Scale-Short Version (subscales: NegU, Negative Urgency; LackP, Lack of Perseverance; LackPre, Lack of Premeditation; SenS, Sensation Seeking; PosU, Positive Urgency); CTQ-SF, short form of Childhood Trauma Questionnaire (subscales: EAb, Emotional Abuse; PAb, Physical Abuse; SAb, Sexual Abuse; ENeg, Emotional Neglect; PNeg, Physical Neglect).

## Data Availability

All data supporting this work will be made freely available via Brunel University London research repository at 10.17633/rd.brunel.25451407.

## Supplementary Materials

The following supporting information can be downloaded at: https://www.mdpi.com/article/10.3390/brainsci14101020/s1, Table S1: Correlations (Pearson’s *r*) between measures of mental health, sleep quality, personality traits and childhood trauma in the UK sample; Table S2: Correlations (Pearson’s *r*) between measures of mental health, sleep quality, personality traits and childhood trauma in German sample.
