# Supplementary material for: Sleep Matters in Chronotype and Mental Health Association: Evidence from the UK and Germany
Source: Brain Sci. 2024 Oct 14;14(10):1020. doi: 10.3390/brainsci14101020 (PMC11505704; doi:10.3390/brainsci14101020)
Supplement: Supplementary file 1 [file brainsci-14-01020-s001.zip › brainsci-3201205-supplementary.pdf]

Supplementary Table S1. Correlations (Pearson's r) between measures of mental health, sleep quality, personality traits and childhood trauma in the UK sample.

	**Mental Health**			**Quality of Sleep**	**Personality Traits**											**Childhood Trauma**				
	**DASS-21**				**EPQ-SF**		**s-OLIFE**				**S-UPPS-P**					**CTQ-SF**				
	**D**	**A**	**S**	**PSQI**	**Extrav**	**Neuro**	**UnEx**	**CogDis**	**IntroAn**	**ImpNn**	**NegU**	**LackP**	**LackPre**	**SenS**	**PosU**	**EAb**	**PAb**	**SAb**	**ENeg**	**PNeg**
D		0.712	0.656	0.565	-0.249	0.478	0.370	0.443	0.358	0.238	0.365	0.063	0.122	-0.114	0.271	0.413	0.199	0.204	0.214	0.183
	1	(<0.001)	(<0.001)	(<0.001)	(<0.001)	(<0.001)	(<0.001)	(<0.001)	(<0.001)	(0.001)	(<0.001)	(0.397)	(0.097)	(0.121)	(<0.001)	(<0.001)	(0.007)	(0.005)	(0.003)	(0.013)
A			0.769	0.535	-0.113	0.570	0.478	0.517	0.314	0.333	0.319	0.059	0.194	-0.166	0.265	0.444	0.285	0.322	0.217	0.291
		1	(<0.001)	(<0.001)	(0.127)	(<0.001)	(<0.001)	(<0.001)	(<0.001)	(<0.001)	(<0.001)	(0.422)	(0.008)	(0.024)	(<0.001)	(<0.001)	(<0.001)	(<0.001)	(0.003)	(<0.001)
S				0.510	-0.071	0.598	0.403	0.506	0.331	0.324	0.361	-0.001	0.213	-0.175	0.269	0.406	0.120	0.268	0.142	0.135
			1	(<0.001)	(0.335)	(<0.001)	(<0.001)	(<0.001)	(<0.001)	(<0.001)	(<0.001)	(0.993)	(0.004)	(0.017)	(<0.001)	(<0.001)	(0.104)	(<0.001)	(0.054)	(0.067)
PSQI					-0.102	0.379	0.236	0.363	0.175	0.203	0.315	0.046	0.136	-0.070	0.175	0.422	0.118	0.230	0.141	0.061
				1	(0.169)	(<0.001)	(0.001)	(<0.001)	(0.017)	(0.006)	(<0.001)	(0.536)	(0.066)	(0.345)	(0.017)	(<0.001)	(0.109)	(0.002)	(0.056)	(0.408)
Extrav						-0.161	-0.082	-0.274	-0.442	0.018	0.060	-0.216	0.004	0.172	0.085	0.020	-0.020	0.159	-0.037	-0.005
					1	(0.029)	(0.268)	(<0.001)	(<0.001)	(0.811)	(0.418)	(0.003)	(0.952)	(0.019)	(0.249)	(0.791)	(0.788)	(0.030)	(0.614)	(0.944)
Neuro							0.524	0.707	0.293	0.335	0.523	-0.018	0.037	-0.235	0.362	0.247	0.080	0.147	0.109	0.126
						1	(<0.001)	(<0.001)	(<0.001)	(<0.001)	(<0.001)	(0.809)	(0.619)	(0.001)	(<0.001)	(<0.001)	(0.281)	(0.046)	(0.138)	(0.086)
UnEx								0.615	0.410	0.500	0.442	-0.061	-0.028	0.033	0.444	0.230	0.167	0.211	0.145	0.243
							1	(<0.001)	(<0.001)	(<0.001)	(<0.001)	(0.411)	(0.710)	(0.659)	(<0.001)	(0.002)	(0.023)	(0.004)	(0.050)	(<0.001)
CogDis									0.362	0.431	0.480	0.109	0.202	-0.103	0.427	0.253	0.056	0.089	0.184	0.125
								1	(<0.001)	(<0.001)	(<0.001)	(0.139)	(0.006)	(0.163)	(<0.001)	(<0.001)	(0.449)	(0.226)	(0.012)	(0.089)
IntroAn										0.263	0.166	0.007	0	-0.179	0.174	0.178	0.128	0.135	0.153	0.170
									1	(<0.001)	(0.024)	(0.929)	(0.999)	(0.015)	(0.018)	(0.016)	(0.083)	(0.067)	(0.038)	(0.021)
ImpNn											0.362	0.085	0.223	0.113	0.439	0.254	0.169	0.211	0.174	0.179
										1	(<0.001)	(0.251)	(0.002)	(0.127)	(<0.001)	(<0.001)	(0.022)	(0.004)	(0.018)	(0.015)
NegU												-0.057	0.119	0.088	0.693	0.240	0.131	0.179	0.084	0.152
											1	(0.438)	(0.108)	(0.235)	(<0.001)	(<0.001)	(0.075)	(0.015)	(0.253)	(0.039)
LackP													0.507	-0.123	0.035	0.059	0.053	-0.072	0.032	0.056
												1	(<0.001)	(0.095)	(0.640)	(0.427)	(0.473)	(0.333)	(0.667)	(0.450)
LackPre														-0.022	0.217	0.181	0.119	0.103	0.093	0.078
													1	(0.767)	(0.003)	(0.014)	(0.108)	(0.163)	(0.206)	(0.292)

SenS		0.197	-0.063	0	0.016	0.113	0.126
	1	(0.007)	(0.392)	(0.998)	(0.828)	(0.127)	(0.087)
PosU			0.148	0.071	0.140	0.139	0.155
		1	(0.045)	(0.34)	(0.057)	(0.060)	(0.035)
EAb				0.669	0.546	0.581	0.432
			1	(<0.001)	(<0.001)	(<0.001)	(<0.001)
PAb					0.430	0.425	0.489
				1	(<0.001)	(<0.001)	(<0.001)
SAb						0.329	0.342
					1	(<0.001)	(<0.001)
ENeg							0.534
						1	(<0.001)
PNeg							1

Abbreviations: DASS-21: Depression Anxiety and Stress Scale-21 (subscales: D, Depression; A, Anxiety; S, Stress); PSQI, Pittsburgh Sleep Quality Index; EPQ-SF, Eysenck Personality Questionnaire-Revised (subscales: Extrav, Extraversion; Neuro, Neuroticism); s-OLIFE, short Oxford-Liverpool Inventory of Feelings and Emotions (subscales: UnEx, Unusual Experience; CogDis, Cognitive Disorganisation; IntroAn, Introvertive Anhedonia; ImpNn, Impulsive Nonconformity); S-UPPS-P, Impulsive Behaviour Scale-Short Version (subscales: NegU, Negative Urgency; LackP, Lack of Perseverance; LackPre, Lack of Premeditation; SenS, Sensation Seeking; PosU, Positive Urgency); CTQ-SF, short form of Childhood Trauma Questionnaire (subscales: EAb, Emotional Abuse; PAb, Physical Abuse; SAb, Sexual Abuse; ENeg, Emotional Neglect; PNeg, Physical Neglect).

Supplementary Table S2. Correlations (Pearson's r) between measures of mental health, sleep quality, personality traits and childhood trauma in German sample.

	Mental Health			Quality of Sleep	Personality Traits											Childhood Trauma				
	DASS-21			PSQI	EPQ-SF		s-OLIFE				S-UPPS-P					CTQ-SF				
	D	A	S		Extrav	Neuro	UnEx	CogDis	IntroAn	ImpNn	NegU	LackP	LackPre	SenS	PosU	EAb	PAb	SAb	ENeg	PNeg
D		0.505	0.620	0.275	-0.193	0.581	0.302	0.508	0.382	0.322	0.337	-0.137	-0.134	0.098	0.293	0.188	0.091	-0.050	0.184	0.108
	1	(<0.001)	(<0.001)	(<0.001)	(0.005)	(<0.001)	(<0.001)	(<0.001)	(<0.001)	(<0.001)	(<0.001)	(0.049)	(0.053)	(0.160)	(<0.001)	(0.006)	(0.193)	(0.471)	(0.008)	(0.120)
A			0.557	0.305	-0.113	0.496	0.535	0.416	0.285	0.281	0.224	-0.039	-0.088	0.019	0.285	0.283	0.189	0.067	0.134	0.127
		1	(<0.001)	(<0.001)	(0.104)	(<0.001)	(<0.001)	(<0.001)	(<0.001)	(<0.001)	(0.001)	(0.571)	(0.205)	(0.784)	(<0.001)	(<0.001)	(0.007)	(0.336)	(0.054)	(0.066)
S				0.271	-0.098	0.687	0.411	0.561	0.190	0.321	0.432	0.002	-0.110	0.032	0.324	0.232	0.079	-0.082	0.170	0.087
			1	(<0.001)	(0.156)	(<0.001)	(<0.001)	(<0.001)	(0.006)	(<0.001)	(<0.001)	(0.973)	(0.113)	(0.648)	(<0.001)	(<0.001)	(0.259)	(0.241)	(0.015)	(0.210)
PSQI					-0.064	0.244	0.308	0.289	0.196	0.186	0.157	0.07	-0.048	0.043	0.121	0.261	0.181	0.188	0.124	0.109
				1	(0.360)	(<0.001)	(<0.001)	(<0.001)	(0.005	(0.007)	(0.024)	(0.318)	(0.49)	(0.534)	(0.082)	(<0.001)	(0.010)	(0.007)	(0.076)	(0.118)
Extrav						-0.225	0.013	-0.233	-0.499	0.183	0.043	0.045	-0.211	0.356	0.153	-0.027	0.011	-0.048	-0.134	-0.01
					1	(0.001)	(0.855)	(<0.001)	(<0.001)	(0.008)	(0.539)	(0.522)	(0.002)	(<0.001)	(0.027)	(0.693)	(0.876)	(0.494)	(0.054)	(0.885)
Neuro							0.402	0.652	0.282	0.288	0.461	-0.07	-0.114	-0.135	0.235	0.213	-0.005	-0.025	0.166	0.004
						1	(<0.001)	(<0.001)	(<0.001)	(<0.001)	(<0.001)	(0.314)	(0.100)	(0.051)	(<0.001)	(0.002)	(0.947)	(0.715)	(0.017)	(0.949)
UnEx								. 0 423	0.182	0.343	0.340	-0.060	-0.159	0.117	0.414	0.271	0.160	0.098	0.117	0.131
							1	(<0.001)	0.008	(<0.001)	(<0.001)	(0.391)	(0.022)	(0.092)	(<0.001)	(<0.001)	(0.023)	(0.159)	(0.095)	(0.059)
CogDis									0.312	0.396	0.470	-0.225	-0.227	-0.003	0.276	0.180	0.068	0.040	0.166	0.079
								1	(<0.001)	(<0.001)	(<0.001)	(0.001)	(<0.001)	(0.969)	(<0.001)	(0.009)	(0.337)	(0.570)	(0.017)	(0.258)
IntroAn										0.083	0.076	-0.052	0.083	-0.088	0.124	0.034	-0.006	-0.012	0.175	0.206
									1	(0.231)	(0.273)	(0.457)	(0.231)	(0.207)	(0.075)	(0.625)	(0.927)	(0.867)	(0.012)	(0.003)
ImpNn											0.444	-0.089	-0.336	0.298	0.386	0.165	0.046	0.064	0.023	0.119
										1	(<0.001)	(0.199)	(<0.001)	(<0.001)	(<0.001)	(0.017)	(0.513)	(0.356)	(0.742)	(0.085)
NegU												-0.138	-0.325	0.158	0.511	0.260	0.026	0.019	0.166	0.06
											1	(0.046)	(<0.001)	(0.022)	(<0.001)	(<0.001)	(0.717)	(0.784)	(0.017)	(0.388)
LackP													0.291	-0.052	-0.044	0.004	0.091	-0.028	-0.036	-0.103
												1	(<0.001)	(0.453)	(0.524)	(0.956)	(0.194)	(0.692)	(0.608)	(0.139)
LackPre														-0.211	-0.210	-0.121	0.058	-0.042	-0.051	-0.027
													1	(0.002)	(0.002)	(0.08)	(0.407)	(0.55)	(0.463)	(0.699)
SenS														1	0.273	0.062	0.025	-0.008	-0.015	-0.008

	(<0.001)	(0.374)	(0.722)	(0.903)	(0.827)	(0.904)
PosU	1	0.093 (0.179)	0.059 (0.402)	0.047 (0.503)	0.049 (0.484)	0.075 (0.279)
EAb		1	0.592 (<0.001)	0.280 (<0.001)	0.644 (<0.001)	0.313 (<0.001)
PAb			1	0.255 (<0.001)	0.419 (<0.001)	0.348 (<0.001)
SAb				1	0.290 (<0.001)	0.263 (<0.001)
ENeg					1	0.537 (<0.001)
PNeg						1

Abbreviations: DASS-21: Depression Anxiety and Stress Scale-21 (subscales: D, Depression; A, Anxiety; S, Stress); PSQI, Pittsburgh Sleep Quality Index; EPQ-SF, Eysenck Personality Questionnaire-Revised (subscales: Extrav, Extraversion; Neuro, Neuroticism); s-OLIFE, short Oxford-Liverpool Inventory of Feelings and Emotions (subscales: UnEx, Unusual Experience; CogDis, Cognitive Disorganisation; IntroAn, Introvertive Anhedonia; ImpNn, Impulsive Nonconformity); S-UPPS-P, Impulsive Behaviour Scale-Short Version (subscales: NegU, Negative Urgency; LackP, Lack of Perseverance; LackPre, Lack of Premeditation; SenS, Sensation Seeking; PosU, Positive Urgency); CTQ-SF, short form of Childhood Trauma Questionnaire (subscales: EAb, Emotional Abuse; PAb, Physical Abuse; SAb, Sexual Abuse; ENeg, Emotional Neglect; PNeg, Physical Neglect).
